# CDK14 regulates the development and repair of lung

**DOI:** 10.1038/s41420-025-02292-4

**Published:** 2025-01-18

**Authors:** Jian-wei Chen, Yu-xiang Wang, Rong-rong Gao, Lan-yue Ma, Jing Zhong, Jia-xin Yang, Zhao-hua Deng, Yu-yan Li, Xiao-ling Li, Ya-hai Shu, Wen-jing Guo, Zi-yuan Zhou, Xiao Yu Tian, Jinjin Ma, Yang Liu, Qi Chen

**Affiliations:** 1 Institutes of physical science and information technology, Anhui University, Hefei, Anhui 230601 China; 2 Center for Cell Lineage Atlas, Guangzhou Institutes of Biomedicine and Health, Chinese Academy of Sciences, Guangzhou, Guangdong 510530 China; 3 University of Chinese Academy of Sciences, Beijing, China; 4 China-New Zealand Belt and Road Joint Laboratory on Biomedicine and Health, Guangdong Provincial Key Laboratory for Stem Cell and Regenerative Medicine, Guangdong-Hong Kong Joint Laboratory for Stem Cell and Regenerative Medicine, CAS Key Laboratory of Regenerative Biology, Joint School of Life Sciences, Guangzhou Institutes of Biomedicine and Health, Chinese Academy of Sciences, Guangzhou Medical University, Guangzhou, Guangdong 510530 China; 5 Biomedical Sciences College & Shandong Medicinal Biotechnology Centre, Shandong First Medical University & Shandong Academy of Medical Sciences; NHC Key Laboratory of biotechnology drugs (Shandong Academy of Medical Sciences); Key Lab for Rare & Uncommon Diseases of Shandong Province, Ji’nan, 250117 Shandong China; 6 The Innovation Centre of Ministry of Education for Development and Diseases, School of Medicine, South China University of Technology, Guangzhou, 510006 China; 7 National Cancer Center/National Clinical Research Center for Cancer/Cancer Hospital & Shenzhen Hospital, Chinese Academy of Medical Sciences and Peking Union Medical College, Shenzhen, 518116 China; 8 CUHK-GIBH CAS Joint Research Laboratory on Stem Cell and Regenerative Medicine, School of Biomedical Sciences, Heart and Vascular Institute, Faculty of Medicine, The Chinese University of Hong Kong, Shatin NT, Hong Kong SAR, 999077 China; 9 The Institute of Future Health, South China University of Technology, Guangzhou International Campus, Guangzhou, 511442 China

**Keywords:** Angiogenesis, Cell proliferation, Development

## Abstract

Cyclin-dependent kinases (CDK) 14 regulates cell cycle, tumor expansion by influencing the downstream targets of the canonical Wnt signaling pathway. However, the function of CDK14 during organ development and regeneration has not been investigated in genetically-modified animals. Here, we found that genetic ablation of *Cdk14* influenced pulmonary vascular endothelial cells and alveolar epithelial cells during mice embryonic development as well as repair of lung after bleomycin or lipopolysaccharide induced injury. Genetic knockout of *Cdk14* and the CDK14 covalent inhibitor FMF-04-159-2 resulted in reduction of pulmonary vessel covered area and epithelial cell number, exhibiting increased mortality and more severe lung damage after injury. Mechanistically, *Cdk14* ablation inhibited the proliferation of epithelial and vascular endothelial cells, inducing cell cycle arrest at the G_2_/M phase. Through RNA-seq analysis of both endothelial and epithelial cells, we found that knockdown of *Cdk14* controls the expression of signal transducers and activator of transcription 1 (STAT1) as well as associated genes in interferon signaling. Disruption of *Cdk14* interferes with IFN-γ induced lung repair in vivo, suggesting potential crosstalk of CDK14 signaling and IFN-γ pathway. Our work highlights the importance of *Cdk14* in lung development and regenerative repair through an uncharacterized CDK14- IFN-γ signaling axis.

## Introduction

The proliferation and mitosis of cells are stringently regulated by genes including cyclin dependent kinases (CDKs) [1]. CDKs are a class of serine/threonine protein kinases, whose level controls the transition of different phases in the cell cycle [2]. The CDK family are currently categorized into eight subfamilies based on amino acid sequence and function. The activation process of CDKs in each subfamily is similar. When cyclin binds to the C-terminal end of CDKs, it activates the ATP-binding site in CDKs, and then ATP enters the catalytic site of CDKs to make serine/threonine phosphorylated allowing CDKs to possess the kinase activity [3]. The different families of CDKs have various functions in the transition process of cell cycle [4]. For example, the binding of CDK4/6 and cyclin D prompts the cell cycle to pass through the checkpoint, which allows the cell to enter the mitosis [5]. During the G1 to S phase transition, binding of CDK2 to Cyclin E phosphorylates the Rb pocket and activates the E2F transcription factor, thereby prompting transcription of downstream genes [6].

CDK14 belongs to the CDK5 subfamily [7], but the function of CDK14 remains largely unexplored. CDK14 is reported to be involved in the canonical Wnt/β-catenin signaling driving the cell cycle into mitosis [8]. CDK14 achieves this by activating intracellular skeleton proteins through phosphorylation and accelerates the formation of spindle filaments during mitosis, thus providing a material basis for the cell to enter mitosis and thus promoting mitosis [9]. CDK14 forms interaction complex with Cyclin Y, and the Wnt signaling receptor protein LRP6 is one of the targets of CDK14/Cyclin Y complex. In G2 phase, Cyclin Y is synthesized and binds to free CDK14 in the cytoplasm, activating CDK14 kinase activity, phosphorylating the PPPSP site of the LRP6 protein, increasing the possibility of CK1 binding to the substrate, and promoting the expression of the Wnt downstream genes, allowing the cell cycle to enter into M phase [10].

*Cdk14* is reported to have higher expression levels in many tumor tissues than in normal tissues, and significantly enhances the proliferation and migration of cancer cells [11, 12]. For example, in osteosarcoma cells, circ-CDK14 directly targets the oncogene miR-198, leading to an increase in the activity of the transcription factor E2F2, which in turn promotes the expression of some oncogenes. By silencing the function of *Cdk14*, the inhibitory effect of miR-198/E2F2 can be weakened, thereby achieving the purpose of tumor treatment [13]. In prostate cancer, it was mentioned that N7-methylguanosine modification after *Cdk14* transcription increases mRNA stability, which in turn contributes to the pathologic growth of prostate cancer. Inhibition of the N7-methylguanosine modifying enzyme METTL1 delayed cancer cell migration and invasive activity due to *Cdk14* overexpression [14]. In 2019, FMF-04-159-2 (FMF) was found to covalently bind to CDK14 [15], which can reduce the activity of breast stem cells and inhibit the progression of triple-negative breast cancer [8]. After in-depth exploration, it was found that the drug, after covalently binding to CDK14, could inhibit the Wnt/β-catenin classical signaling pathway, which could inhibit the clone formation of mammary basal cells and the invasive ability of breast cancer cells. Therefore, it is a feasible strategy to use CDK14 as a therapeutic target and inhibit tumors in some *Cdk14* overexpressed tumor types.

However, most of current researches regarding *Cdk14* rely heavily on shRNA-mediated knockdown or pharmacological reagents [16]. The off-target effects of these methodologies are inevitable. *Cdk14* genetic knockout mice have not been utilized to investigate the function of this gene during development and regeneration of living mammals. In addition, it remains largely unknown whether CDK14 is involved in other signaling except canonical Wnt pathway.

In this work, we found that CDK14 regulates the embryonic development of lung by using *Cdk14* knockout mice. Genetic and pharmacological ablation indicates that CDK14 is indispensable during the regeneration and repair of bleomycin and lipopolysaccharide (LPS) induced lung injury. Mechanistically, we found that CDK14 influences the expression of signal transducers and activator of transcription 1 (STAT1) in endothelial and epithelial cells. Therefore, the presence of *Cdk14* is indispensable in the response of lung to interferon-gamma (IFN-γ) induced alveolar repair.

## Results

### Cdk14 regulates embryonic development of lung

To investigate the function of *Cdk14* in genetically-modified animals, we removed the exon 3 of *Cdk14* to generate the *Cdk14* knockout mice (*Cdk14*^−/−^) (Fig. S1A). This was because exon 3 includes the ATP-binding site and a serine/threonine kinase active site of CDK14. Genetical removal of exon 3 eliminated 95 base pairs in the coding sequence of *Cdk14* to induce a shift of reading frame to disrupt the function of CDK14. Based on the genotyping results, we calculated the proportions of the offsprings produced by mating the heterozygous parents. In a total of 105 mice, we obtained 26 wild-type, 55 heterozygous, and 24 homozygous knockout mice (Fig. S1B). This result indicated that the offspring proportions were consistent with Mendel’s laws of inheritance, and therefore the *Cdk14* homozygous knockout mice did not result in direct death of mice.

**Fig. 1:**
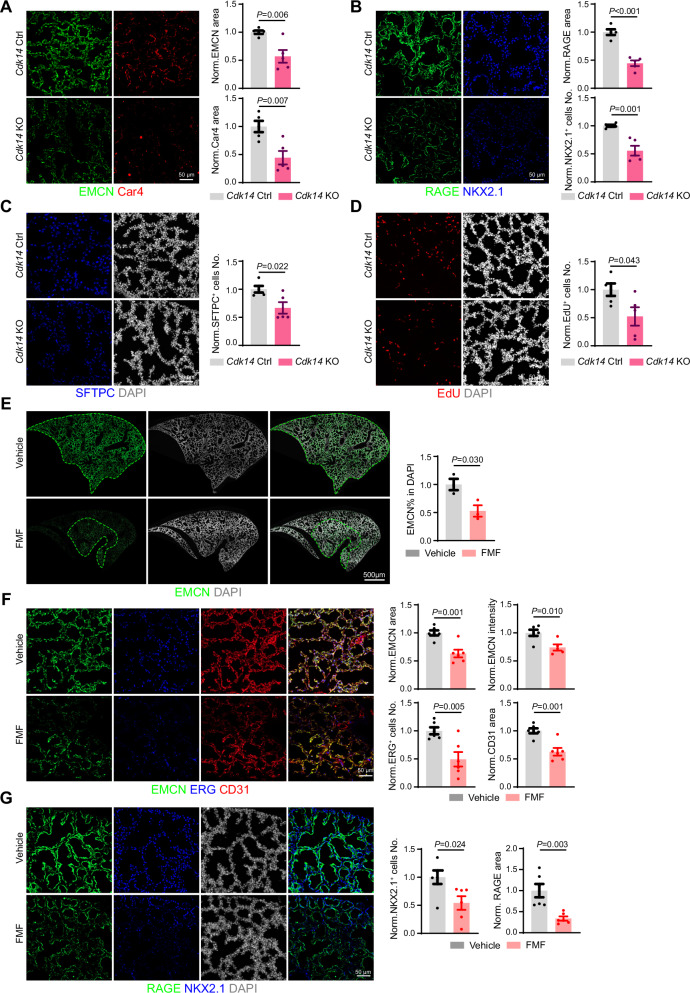
*Cdk14* regulates lung embryonic development. **A** Representative projected confocal images showing EMCN^+^ (green) and Car4^+^ (red) vascular endothelial cells in *Cdk14*^*+/+*^ or *Cdk14*^*−/−*^ E19.0 lungs. Quantification about average intensity and covered area of EMCN^+^ blood vessels and intensity of Car4^+^ Cells. All data are normalized to *Cdk14*^+/+^. *Cdk14*^+/+^=5；*Cdk14*^−/−^=5. Error bars, mean $\pm$ s.e.m. *P* values, *t*-test. **B** Representative projected confocal images showing NKX2.1^+^ (blue) lung epithelial progenitor cells, RAGE^+^ (green) AT1 in *Cdk14*^+/+^ or *Cdk14*^−/−^ E19.0 lungs. Quantification about average intensity and covered area of RAGE labelled AT1 and NKX2.1 labelled lung epithelial progenitor cell number in *Cdk14*^*+/+*^ or *Cdk14*^*−/−*^ E19.0 lungs. All data are normalized to *Cdk14*^+/+^. *Cdk14*^+/+^=5；*Cdk14*^−/−^=5. Error bars, mean $\pm$ s.e.m. *P* values, *t*-test. **C** Representative projected confocal images showing SFTPC^+^ (blue) AT2 progenitor cells. Quantification about SFTPC labelled AT2 cell number in *Cdk14*^*+/+*^ or *Cdk14*^*−/−*^ E19.0 lungs. All data are normalized to *Cdk14*^+/+^. *Cdk14*^+/+^=5；*Cdk14*^−/−^=5. Error bars, mean $\pm$ s.e.m. *P*-values, *t*-test. **D** Representative projected confocal images showing EdU^+^ cells, Quantification about proliferating cells in *Cdk14*^*+/+*^ or *Cdk14*^*−/−*^ E19.0 lungs. All data are normalized to *Cdk14*^+/+^. *Cdk14*^+/+^=5；*Cdk14*^−/−^=5. Error bars, mean $\pm$ s.e.m. P values, t-test. **E** Representative projected confocal images showing area of EMCN^+^ blood vessels and DAPI in FMF-treated or vehicle treated E19.0 lung. Quantification about EMCN percentage to DAPI. All data are normalized to vehicle. Vehicle = 3；FMF = 3. Error bars, mean $\pm$ s.e.m. *P* values, *t*-test. **F** Representative projected confocal images showing EMCN, ERG and CD31 in FMF-treated or vehicle treated E19.0 lung. Quantification about average intensity and covered area of EMCN, ERG labelled endothelial cell number and covered area of CD31 in FMF-treated or vehicle treated E19.0 lung. All data are normalized to vehicle. Vehicle = 6；FMF = 5 ~ 6. Error bars, mean $\pm$ s.e.m. *P* values, *t*-test. **G** Representative projected confocal images showing RAGE, NKX2.1 and DAPI in FMF-treated or vehicle treated E19.0 lung. Quantification about covered area of RAGE and NKX2.1 labelled lung epithelial progenitor cell number in FMF-treated or vehicle treated E19.0 lung. All data are normalized to vehicle. Vehicle = 6；FMF = 6. Error bars, mean $\pm$ s.e.m. *P* values, *t*-test.

By searching Tabula Muris, a public single cell RNA-sequencing database for whole organ of mice [17], we noted that *Cdk14* was not ubiquitously expressed in every organ and cell of mice but was relatively enriched in the lung (Fig. S1C–S1E). To investigate in depth the effect of *Cdk14* knockout during lung development, we performed immunofluorescent staining to visualize the morphogenesis of lung endothelial cells and epithelial cells. In embryonic lung, the expression of vascular endothelial cell marker (EMCN) as well as carbonic anhydrase 4^+^ (Car4^+^) capillaries were significantly reduced in *Cdk14* knockout mice comparing with littermate control (Fig. 1A) [18]. Similarly, the advanced glycosylation end product-specific receptor (RAGE) labeled type 1 alveolar epithelial cells (AT1), the expression of NK2 homeobox 1 (NKX2.1), a protein used to label lung epithelial progenitor cells, as well as the number of surface-active protein C (SFTPC)-labeled type 2 alveolar epithelial cells (AT2) showed trends of decreasing (Fig. 1B, C). When 5-Ethynyl-2’-deoxyuridine (EdU) was incorporated to label the proliferating cells in the lung, we observed a defective EdU positive cell number in *Cdk14* knockout mice (Fig. 1D). These evidences indicated that genetic knockout of *Cdk14* resulted in endothelial and epithelial cell developmental defects together with proliferation delay in lung.

To confirm these developmental defects in *Cdk14* knockout mice, we made use of a CDK14 antagonist, namely FMF-04-159-2 (hereinafter abbreviated as FMF), which covalently binds to the CDK14 protein with a selective inhibitory effect on the progression of triple-negative breast cancer [8]. When this CDK14 inhibitor was injected into the wildtype mice, we detected a significant reduction in EMCN -covered vascularized area in embryonic lung (Fig. 1E). In the vascularized area, FMF treatment resulted in significant reduction in the expression of EMCN, ERG and CD31, (Fig. 1F). Similarly, the number of NKX2.1^+^ lung epithelial progenitor cells and the expression of AT1 cell marker RAGE were significantly reduced after FMF treatment (Fig. 1G). These developmental defects continued until adult with alteration of Emcn-labelled vascular area, RAGE^+^ AT1 epithelial cell area as well as the numbers of SFTPC^+^ and NKX2.1^+^ epithelial cell in *Cdk14* knockout mice (Fig. S2).

These evidences suggest that genetic and pharmacological ablation of CDK14 influence endothelial and epithelial cell development in lung.

### Cdk14 is indispensable during lung repair after damage

**Fig. 2:**
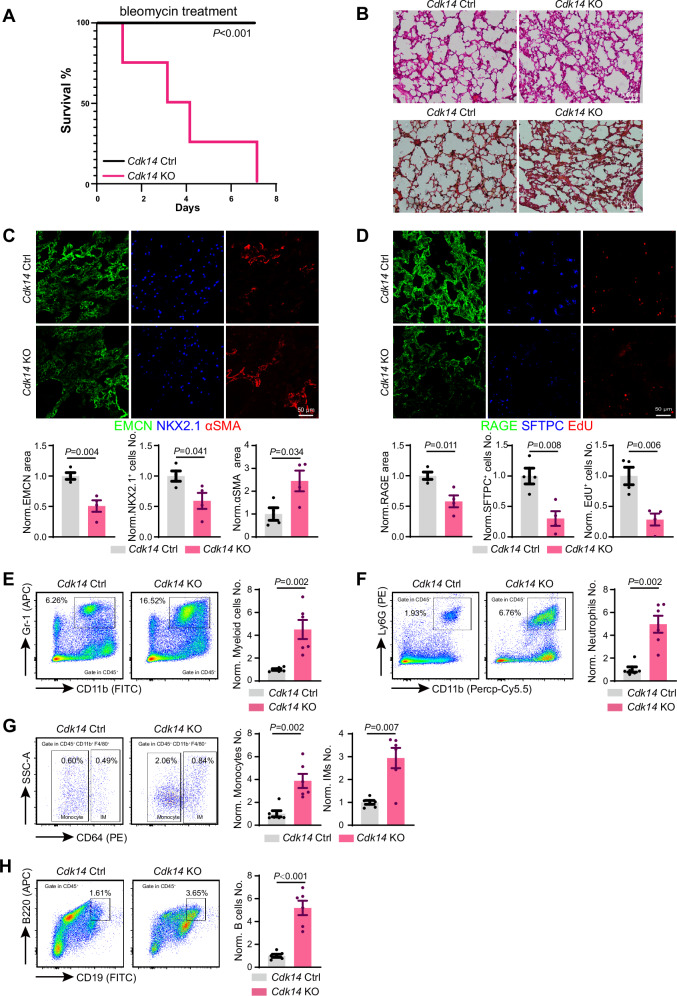
*Cdk14* is indispensable during bleomycin-induced lung regeneration. **A** Survival curve of *Cdk14*^*+/+*^ (*n* = 4) and *Cdk14*^*−/−*^ (*n* = 4) after intratracheal injection of 0.3 mg/ml bleomycin 50 μl. Log-rank (Mantel-Cox) test was used to calculate the *P* value. **B** Representative histological morphology of *Cdk14*^+/+^ and *Cdk14*^−/−^ mice lung stained by hematoxylin-eosin and sirius red. **C** Representative projected confocal images showing EMCN^+^ (green) blood vessels and NKX2.1^+^ (blue) lung epithelial progenitor cell and αSMA (red) labelled myofibroblasts in bleomycin-treated *Cdk14*^+/+^ and *Cdk14*^−/−^ mice lung. Quantification about covered area of EMCN and αSMA and NKX2.1 labelled lung epithelial progenitor cell number in bleomycin-treated *Cdk14*^+/+^ and *Cdk14*^−/−^ mice lung. All data are normalized to *Cdk14*^+/+^. *Cdk14*^+/+^ = 4；*Cdk14*^−/−^ = 4. Error bars, mean $\pm$ s.e.m. *P* values, *t*-test. **D** Representative projected confocal images showing RAGE^+^ (green), SFTPC+ (blue) and EdU^+^ cells in bleomycin-treated *Cdk14*^+/+^ and *Cdk14*^−/−^ mice lung. Quantification about covered area of RAGE and SFTPC labelled AT2 cell number in bleomycin-treated *Cdk14*^+/+^ and *Cdk14*^−/−^ mice lung. All data are normalized to *Cdk14*^+/+^. *Cdk14*^+/+^ = 4；*Cdk14*^−/−^ = 4. Error bars, mean $\pm$ s.e.m. *P* values, *t*-test. **E** Diagram depicting for CD11b^+^Gr-1^+^ cells in bleomycin-treated ctrl and *Cdk14* KO mice lung. Quantification about the number of CD11b^+^Gr-1^+^ myeloid cells in the two groups. Cell frequencies show the percentage of target population in total cells. *Cdk14* Ctrl = 6；*Cdk14* KO = 6. Error bars, mean ± s.e.m. *P* values, *t*-test. **F** Diagram depicting for neutrophils (Ly6G^+^, CD11b^+^) in bleomycin-treated ctrl and *Cdk14* KO mice lung. Quantification about the number of neutrophils in the two groups. Cell frequencies show the percentage of target population in total cells. *Cdk14* Ctrl = 6；*Cdk14* KO = 6. Error bars, mean ± s.e.m. *P* values, *t*-test. **G** Diagram depicting for interstitial macrophages (CD64^+^) and monocytes (CD64^-^) in bleomycin-treated ctrl and *Cdk14* KO mice lung. Quantification about the number of interstitial macrophages and monocytes in the two groups. Cell frequencies show the. percentage of target population in total cells. *Cdk14* Ctrl = 6；*Cdk14* KO = 6. Error bars, mean ± s.e.m. *P*-values, *t*-test. **H** Diagram depicting for B cells (B220^+^, CD19^+^) in bleomycin-treated ctrl and *Cdk14* KO mice lung. Cell frequencies show the percentage of target population in total cells. Quantification about the number of B cells in the two groups. *Cdk14* Ctrl = 6；*Cdk14*. KO = 6. Error bars, mean ± s.e.m. *P* values, *t*-test.

Next, we investigated the necessity of CDK14 during the regeneration and repair of lung after damage that request active proliferation of cells. Firstly, we utilized the well-established bleomycin-induced lung injury model by intratracheal injection of bleomycin into the mice. Under the normal bleomycin dosage, all the littermate control mice survived but none of the *Cdk14* knockout mice can tolerate more than 7 days after bleomycin treatment (Fig. 2A). To evaluate how CDK14 influenced the lung regeneration after bleomycin damage, we used a lower dosage of bleomycin to guarantee the survival of *Cdk14*^−/−^ mice. Under this condition, we detected a thicker cellular wall visualized by immunohistochemistry tissue staining (Fig. 2B). Immunofluorescent staining indicated the EMCN^+^ alveolar blood vessel covered area was reduced together with higher alveolar area stained by αSMA, which is considered an essential marker for fibrosis (Fig. 2C). In the epithelial cells, we detected a significant reduction of NKX2.1^+^ progenitor cells (Fig. 2C), SFTPC^+^ AT2-type alveolar epithelial cells, as well as a reduction of RAGE^+^ AT1-type alveolar epithelial cells (Fig. 2D). In addition, EdU incorporation experiment during bleomycin-induced lung injury model showed similar proliferating defects after *Cdk14* ablation (Fig. 2D). In addition to endothelial and epithelial cell changes, FACS analysis of leukocyte subpopulation detected higher percentage CD45^+^CD11b^+^Gr-1^+^ myeloid cells (Fig. 2E), CD45^+^CD11b^+^Ly6G^+^ neutrophil (Fig. 2F), CD45^+^CD11b^+^CD11c^low/-^F4/80^+^CD64^+^ interstitial macrophage and CD45^+^CD11b^+^ CD11c^low/-^F4/80^+^CD64^-^ monocyte (Fig. 2G), indicating most of myeloid lineage immune cell was increased in *Cdk14* knockout mice after bleomycin damage, which may suggest a prolonged inflammatory condition. In lymphocyte subpopulation, the CD45^+^CD19^+^B220^+^ B-lymphocyte was increased (Fig. 2H). To confirm the functional role of CDK14 after bleomycin treatment, we injected FMF together with bleomycin that resulted in similar phenotype including cellular wall thickness, fibrosis, reduced EMCN area and reduction of NKX2.1^+^ cell number, SFTPC^+^ cell number and RAGE^+^ AT1 cell (Fig. S3).

**Fig. 3:**
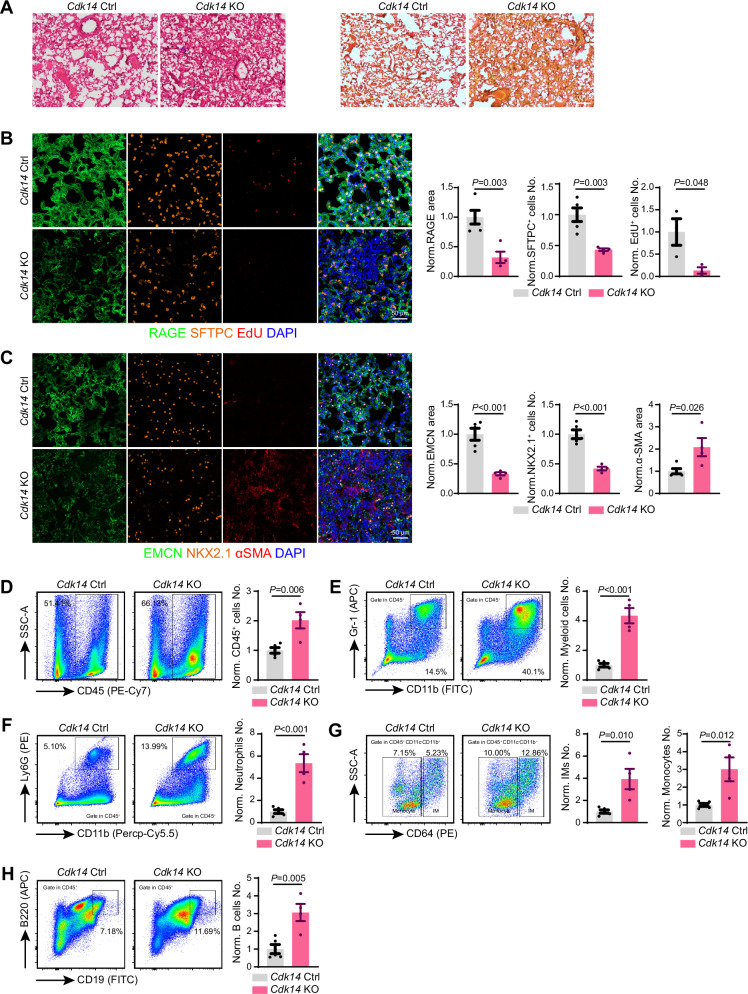
*Cdk14* ablation inhibits the recovery from LPS-induced lung injury. **A** Representative histological morphology of hematoxylin-eosin and sirius red staining of lung from *Cdk14* Ctrl and *Cdk14* KO mice following LPS-induced lung injury. **B** Representative projected confocal images showing RAGE^+^ (green), SFTPC^+^ (orange) and EdU^+^ (red) cells in LPS-treated *Cdk14* Ctrl and *Cdk14* KO mice lung. Quantification about covered area of RAGE, SFTPC labelled AT2 cell number and EdU+ cell number in LPS-treated *Cdk14* Ctrl and *Cdk14* KO mice lung. All data are normalized to *Cdk14* Ctrl. *Cdk14* Ctrl = 3 ~ 5；*Cdk14* KO = 3 ~ 4. Error bars, mean $\pm$ s.e.m. *P* values, *t*-test. **C** Representative projected confocal images showing EMCN^+^ (green) blood vessels and NKX2.1^+^ (orange) lung epithelial progenitor cell and αSMA (red) labelled myofibroblasts in LPS-treated *Cdk14* Ctrl and *Cdk14* KO mice lung. Quantification about covered area of EMCN and αSMA and NKX2.1 labelled lung epithelial progenitor cell number in LPS-treated *Cdk14* Ctrl and *Cdk14* KO mice lung. All data are normalized to *Cdk14* Ctrl. *Cdk14* Ctrl = 5；*Cdk14* KO = 4. Error bars, mean $\pm$ s.e.m. *P* values, *t*-test. **D** Diagram depicting for CD45^+^ cells in LPS-treated *Cdk14* Ctrl and *Cdk14* KO mice lung. Quantification about the number of CD45^+^ cells in the two groups. Cell frequencies show the percentage of target population in total cells. *Cdk14* Ctrl = 5；*Cdk14* KO = 4. Error bars, mean $\pm$ s.e.m. *P* values, *t*-test. **E** Diagram depicting for myeloid cells (Gr-1^+^, CD11b^+^) in LPS-treated *Cdk14* Ctrl and *Cdk14* KO mice lung. Quantification about the number of myeloid cells in the two groups. Cell frequencies show the percentage of target population in total cells. *Cdk14* Ctrl = 5；*Cdk14* KO = 4. Error bars, mean $\pm$ s.e.m. *P* values, *t*-test. **F** Diagram depicting for neutrophils (Ly6G^+^, CD11b^+^) in LPS-treated *Cdk14* Ctrl and *Cdk14* KO mice lung. Quantification about the number of neutrophils in the two groups. Cell frequencies show the percentage of target population in total cells. *Cdk14* Ctrl = 5；*Cdk14* KO = 4. Error bars, mean $\pm$ s.e.m. *P* values, *t*-test. **G** Diagram depicting for interstitial macrophages (CD64^+^) and monocytes (CD64^-^) in LPS-treated *Cdk14* Ctrl and *Cdk14* KO mice lung. Quantification about the number of interstitial macrophages and monocytes in the two groups. Cell frequencies show the percentage of target population in total cells. *Cdk14* Ctrl = 5；*Cdk14* KO = 4. Error bars, mean $\pm$ s.e.m. *P* values, *t*-test. **H** Diagram depicting for B cells (B220^+^, CD19^+^) in LPS-treated *Cdk14* Ctrl and *Cdk14* KO mice lung. Cell frequencies show the percentage of target population in total cells. Quantification about the number of B cells in the two groups. *Cdk14* Ctrl = 5；*Cdk14* KO = 4. Error bars, mean $\pm$ s.e.m. *P* values, *t*-test.

To examine whether CDK14 was necessary for different types of lung injury, we intratracheally injected LPS, an essential component of Gram-negative bacterial and one of the most frequently used pathogens, to induce acute lung injury. Similar to bleomycin, *Cdk14* knockout mice showed defects in EMCN^+^ lung vascularized area as well as NKX2.1^+^, SFTPC^+^, RAGE^+^ epithelial cell number and area (Fig. 3A–C). EdU incorporation experiments displayed similar proliferation delay together with more severe αSMA^+^ fibrosis during LPS-induced lung repair in *Cdk14* knockout mice (Fig. 3B, C). These evidences indicated that absence of CDK14 generated very similar alveolar defects when different chemicals were used to induce lung repair after damage. Furthermore, because LPS was well-known to induce inflammatory response of infected tissue [19], we similarly checked the immune cell number after LPS damage. In *Cdk14* knockout mice, FACS analysis detected higher percentage of CD45^+^ total leukocyte (Fig. 3D), myeloid cells (Fig. 3E), neutrophil (Fig.3F), interstitial macrophage and monocyte (Fig. 3G), indicating a similar myeloid lineage increase to bleomycin-induced lung damage. In lymphocyte subpopulation, the CD19^+^B220^+^ B-lymphocyte was increased but CD4^+^ and CD8^+^ T-lymphocyte was not dramatically changed (Fig. 3H and S4A). To understand why leukocyte was higher after bleomycin or LPS damage in *Cdk14* knockout mice, we used sublethal irradiation to remove some of endogenous leukocytes and transplant GFP^+^ bone marrow cells from *Vav1-Cre Rosa26-mTmG* donor mice to trace engraftment potential of leukocyte in peripheral blood. In *Cdk14* knockout mice, we detected significantly higher GFP^+^ leukocyte and myeloid cell number in the lung (Fig.S4B, S4C). FACS-sorting myeloid cells into vascular barrier formed by control or *shCdk14* endothelial cells showed that more myeloid cells could cross *shCdk14* vascular barrier in vitro (Fig.S4D, S4E). These evidences suggested that CDK14 removal disrupted the vascular barrier function of endothelial cells in vivo and in vitro.

Taken together, these data suggested that CDK14 is indispensable during bleomycin and LPS induced lung injury and repair.

### Cdk14 influences the proliferation and migration of endothelial and epithelial cells in vitro

**Fig. 4:**
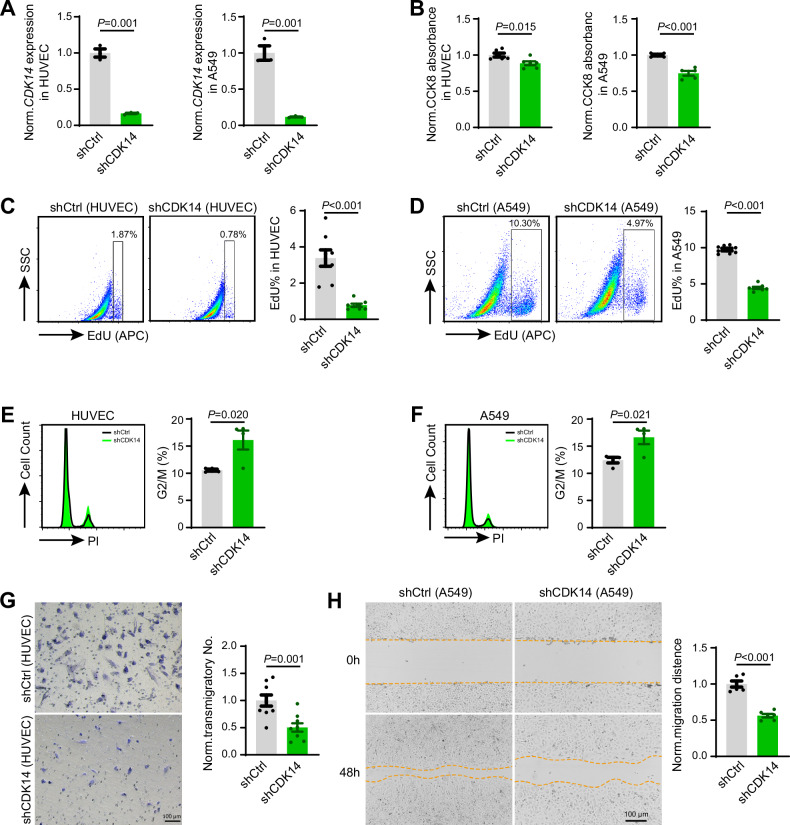
*Cdk14* influences the proliferation and migration of endothelial and epithelial cells in vitro. **A** RT-PCR showing the intracellular mRNA levels of *Cdk14* in HUVECs and A549 cells treated with shCDK14. All data are normalized to shCtrl. shCtrl = 3；shCDK14 = 3. Error bars, mean $\pm$ s.e.m. *P* values, *t*-test. **B** CCK-8 assays were performed to show the effect of shCDK14 on hampered proliferation in HUVECs or A549 cells. All data are normalized to shCtrl. shCtrl = 5 ~ 6；shCDK14 = 5 ~ 6. Error bars, mean $\pm$ s.e.m. *P* values, *t*-test. **C** HUVECs were infected with shCDK14 to knockdown *Cdk14*. Flow cytometry analysis of HUVEC proliferative ability. All data are normalized to shCtrl. shCtrl = 8；shCDK14 = 8. Error bars, mean $\pm$ s.e.m. *P* values, *t*-test. **D** A549 cells were infected with shCDK14 to knockdown *Cdk14*. Flow cytometry analysis of A549 cells proliferative ability. All data are normalized to shCtrl. shCtrl = 8；shCDK14 = 8. Error bars, mean $\pm$ s.e.m. *P* values, *t*-test. **E** PI staining assays were performed to show the percentage of G2/M phase in HUVECs. All data are normalized to shCtrl. shCtrl = 4；shCDK14 = 4. Error bars, mean $\pm$ s.e.m. *P* values, *t*-test. **F** PI staining assays were performed to show the percentage of G2/M phase in A549 cells. All data are normalized to shCtrl. shCtrl = 4；shCDK14 = 4. Error bars, mean $\pm$ s.e.m. *P* values, *t*-test. **G** HUVECs were infected with shCDK14 to knockdown *Cdk14*, after 96 h of infection, transwell assays were performed, and pictures were taken 16 h later. All data are normalized to shCtrl. shCtrl = 9；shCDK14 = 9. Error bars, mean $\pm$ s.e.m. *P* values, *t*-test. **H** A549 cells were infected with shCDK14 to knockdown *Cdk14*, after 96 h of infection, scratch assays were performed, and pictures were taken in 0 h and 48 h. All data are normalized to shCtrl. shCtrl = 6；shCDK14 = 6. Error bars, mean $\pm$ s.e.m. *P* values, *t*-test.

After observing that *Cdk14* knockout resulted in abnormal development and repair of pulmonary vascular endothelial cells and epithelial cells in vivo, we next investigated how CDK14 influences the behavior of cells on human umbilical vein endothelial cells (HUVECs) and A549 epithelial cells in vitro. To achieve this, we constructed shRNAs targeting human *CDK14* gene and transfected the HUVEC and A549 via lentivirus-mediated infection. This strategy was very effective to knockdown *CDK14* with an efficiency higher than 75% (Fig. 4A and S5A). Consistent with our previous in vivo data, EdU incorporation experiment and cell counting kit-8 (CCK8) absorbance assay indicated reduction of cell proliferation in HUVECs and A549 cells after *CDK14* knockdown (Fig. 4B–D). To understand the defective cell cycle phase after *CDK14* knockdown, we stained the propidium iodide to quantify the DNA content, in which we found that the percentage of cells in the tetraploid state (4n), an indication of G_2_/M phase, was higher in *CDK14* knockdown group (Fig. 4E, F). Because *CDK14* knockdown resulted in lower EdU incorporation and CCK8 absorbance, namely reduction of cell proliferation, therefore this result reflected shCDK14 knockdown induced cell cycle arrest at the G_2_/M phase in both HUVECs and A549 cells, which is consistent with previous knowledge in tumor cells [10].

Next, we investigated whether CDK14 influenced other behavior of cells. We found that knockdown of *Cdk14* within either murine or human vascular endothelial cell lines significantly inhibited the migratory capacity of endothelial cells (Fig. 4G and S5B–S5D). Similarly, the migratory area was significantly reduced after knockdown of *CDK14* in A549 epithelial cells (Fig. 4H). These evidences indicated that *CDK14* knockdown influenced the proliferation and migration of both endothelial and epithelial cells.

### Cdk14 knockdown changes the transcriptomic profiles of cells

**Fig. 5:**
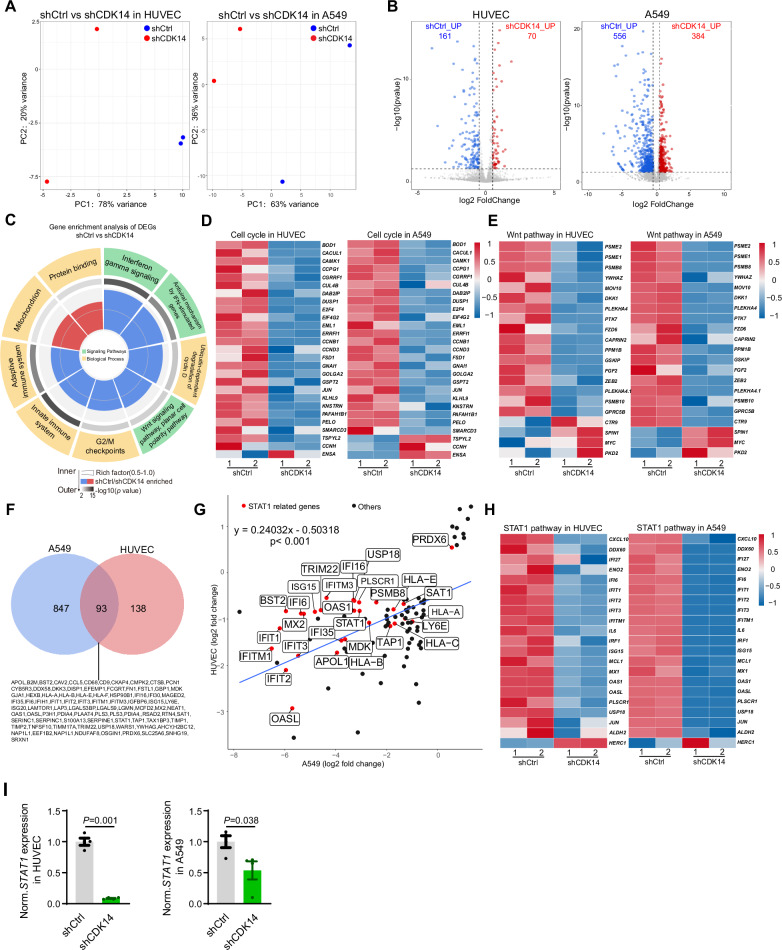
*Cdk14* knockdown changes the transcriptome of endothelial and epithelial cells. **A** Principal component analysis of two datasets (shCtrl, shCDK14) in HUVECs or A549 cells. Note that two datasets have different genes expression, and samples in the same group have little difference both in HUVEC and A549. **B** Volcano plot analysis to compare the variance of two datasets (shCtrl, shCDK14) in HUVECs and A549 cells. **C** Gene ontology (GO) analysis of the biological process and signaling pathways enriched in shCtrl or shCDK14. **D** Heat map of selected cell cycle pathway in shCtrl and shCDK14 group. **E** Heat map of selected cell Wnt pathway in shCtrl and shCDK14 group. **F** Venn diagram was used to analyze the common DEGs between HUVEC and A549. **G** Related analysis shows the correlation of common DEGs with the *STAT1* gene. **H** Heat map of selected STAT1 pathway in shCtrl and shCDK14 group. **I** RT-PCR showing mRNA levels of STAT1 in HUVECs and A549 cells treated with shCDK14. All data are normalized to shCtrl. shCtrl = 4；shCDK14 = 4. Error bars, mean $\pm$ s.e.m. *P* values, *t*-test.

Previous studies indicated that CDK14 is associated with the canonical Wnt signaling pathway by inhibiting the formation of intracytoplasmic β-catenin destruction complexes [20]. It remains largely unknown whether CDK14 influenced other signaling pathways. To explore the downstream target of CDK14 through an unbiased analysis, we performed RNA-sequencing analysis in both HUVEC and A549 cells after shRNA-mediated *CDK14* knockdown. Principal component analysis indicated the primary principal component (PC1) can clearly separate control and *CDK14* knockdown group in both HUVEC and A549 (Fig. 5A). Differentially-expressed gene (DEG) analysis identified more than 200 and 900 DEGs in HUVECs and A549 cells, respectively (Fig. 5B).

Because HUVECs and A549 cells exhibited similar proliferative and migrative defects after *CDK14* knockdown, we compared the common signaling pathway in gene ontology analysis of these DEGs. As expected, this analysis found pathways related with G2/M checkpoint and Wnt signaling (Fig. 5C). Consistently, we found genes related with cell cycle including BOD1, JUN, CAMK1, DUSP1, E2F4, CCNB1, CCND3 and CCNH were significantly down-regulated after *CDK14* knockdown in both HUVEC and A549 (Fig. 5D). We also observed that the expression of some genes in Wnt signaling pathway, such as DKK1, FZD6, MYC and GSKIP, were changed when *CDK14* were knockdown (Fig. 5E). These evidences indicated that our analysis was largely consistent with previous knowledge about CDK14 signaling [21].

Unexpectedly, we noticed that signaling related with immune system especially interferon signaling were changed when *CDK14* were knockdown (Fig. 5C). Interferon (IFN) signaling is an essential pathway in the immune and inflammatory response [22]. The binding of interferons with their corresponding receptors resulted in phosphorylation of JAK-STAT to induce expression of interferon regulated genes [23], but the association between CDK14 and IFN signaling is not reported. We noticed that a large number of genes involved in IFN-STAT1 signaling were changed in both HUVEC and A549 cell (Fig. 5F). To statistically quantify this, we identified 93 common downstream targets of *CDK14*, which were shared in both endothelial and epithelial cells (Fig. 5F, G). In the common DEGs of endothelial and epithelial, we noted that a large number of genes in the IFN signaling, which was most likely because the expression of STAT1, a central regulator of IFN signaling, was down-regulated when *CDK14* were knockdown (Fig. 5G, H). Quantitative PCR analysis validated that the transcriptions of *STAT1* were down-regulated after *CDK14* knockdown (Fig. 5I).

These evidences suggested that *CDK14* knockdown significantly changed the transcription profiles in both endothelial and epithelial cells, resulting in the alteration of common downstream targets including uncharacterized IFN-STAT1 signaling.

### Cdk14 influences IFN-γ induced lung repair in vivo

IFN-γ, as the sole member of mammalian type II IFN, is a multifunctional cytokine which exerts its effects through activating STAT1 to participate in both innate and adaptive immunity [24]. IFN-γ is reported to be involved in lung regeneration and repair after virus infection [25]. Therefore, we used *Cdk14* knockout mice to investigate the potential crosstalk of CDK14 and IFN-γ signaling during LPS-induced acute lung injury model.

**Fig. 6:**
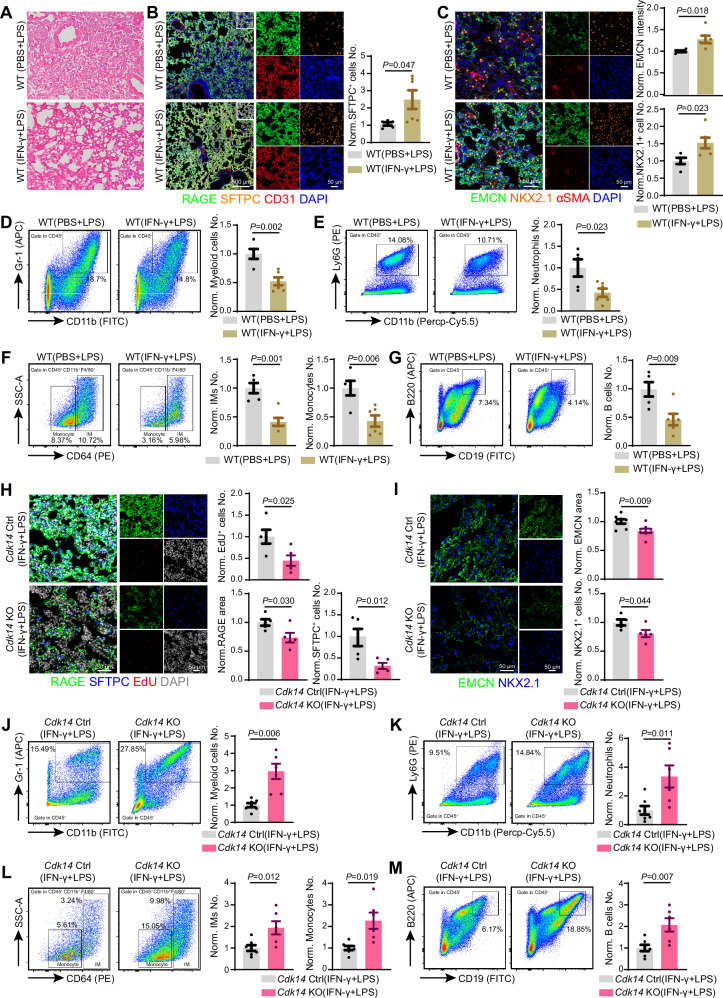
*Cdk14* participates in IFN-γ induced lung repair. **A** Representative histological morphology of lungs from mice with LPS induced injury, treated with PBS or IFN-γ, and stained by hematoxylin-eosin. **B** Representative projected confocal images showing RAGE (green), SFTPC (orange) and CD31 (red) in lungs from mice with LPS induced injury, treated with PBS or IFN-γ. Quantification about SFTPC labelled AT2 cell number in in the two groups. All data are normalized to WT (PBS + LPS). WT (PBS + LPS) = 5；WT (IFN-γ + LPS) = 6. Error bars, mean $\pm$ s.e.m. *P* values, *t*-test. **C** Representative projected confocal images showing EMCN (green), NKX2.1 (orange) and αSMA (red) in lungs from mice with LPS induced injury, treated with PBS or IFN-γ. Quantification about NKX2.1 labelled lung epithelial progenitor cell number and the intensity of EMCN in the two groups. All data are normalized to WT (PBS + LPS). WT (PBS + LPS) = 5；WT (IFN-γ + LPS) = 6. Error bars, mean $\pm$ s.e.m. *P* values, *t*-test. **D** Diagram depicting for myeloid cells (Gr-1^+^, CD11b^+^) in lungs from mice with LPS induced injury, treated with PBS or IFN-γ. Quantification about the number of myeloid cells in the two groups. WT (PBS + LPS) = 5；WT (IFN-γ + LPS) = 6. Error bars, mean $\pm$ s.e.m. *P* values, *t*-test. **E** Diagram depicting for neutrophils (Ly6G^+^, CD11b^+^) in lungs from mice with LPS induced injury, treated with PBS or IFN-γ. Quantification about the number of neutrophils in the two groups. Cell frequencies show the percentage of target population in total cells. WT (PBS + LPS) = 5；WT (IFN-γ + LPS) = 6. Error bars, mean $\pm$ s.e.m. *P* values, *t*-test. **F** Diagram depicting for interstitial macrophages (CD64^+^) in lungs from mice with LPS induced injury, treated with PBS or IFN-γ. Quantification about the number of interstitial macrophages and monocytes in the two groups. Cell frequencies show the percentage of target population in total cells. WT (PBS + LPS) = 5；WT (IFN-γ + LPS) = 6. Error bars, mean $\pm$ s.e.m. *P* values, *t*-test. **G** Diagram depicting for B cells (B220^+^, CD19^+^) in lungs from mice with LPS induced injury, treated with PBS or IFN-γ. Quantification about the number of B cells in the two groups. Cell frequencies show the percentage of target population in total cells. WT (PBS + LPS) = 5；WT (IFN-γ + LPS) = 6. Error bars, mean $\pm$ s.e.m. *P* values, *t*-test. **H** Representative projected confocal images showing RAGE (green), SFTPC (blue) and EdU (red) in lungs of *Cdk14* Ctrl and *Cdk14* KO mice following co-injection of LPS and IFN-γ. Quantification about covered area of RAGE, SFTPC labelled AT2 cell number and EdU+ cells in the two groups. All data are normalized to *Cdk14* Ctrl (IFN-γ + LPS). *Cdk14* Ctrl (IFN-γ + LPS) = 5；*Cdk14* KO (IFN-γ + LPS) = 5. Error bars, mean $\pm$ s.e.m. *P* values, *t*-test. **I** Representative projected confocal images showing Emcn (green) and NKX2.1 (blue) in lungs of *Cdk14* Ctrl and *Cdk14* KO mice following co-injection of LPS and IFN-γ. *Cdk14* Ctrl (IFN-γ + LPS) = 5；*Cdk14* KO (IFN-γ + LPS) = 5. Error bars, mean $\pm$ s.e.m. *P* values, *t*-test. **J** Diagram depicting for myeloid cells (Gr-1^+^, CD11b^+^) in lungs of *Cdk14* Ctrl and *Cdk14* KO mice following co-injection of LPS and IFN-γ. Quantification about the number of myeloid cells in the two groups. Cell frequencies show the percentage of target population in total cells. *Cdk14* Ctrl (IFN-γ + LPS) = 7；*Cdk14* KO (IFN-γ + LPS) = 6. Error bars, mean $\pm$ s.e.m. *P* values, *t*-test. **K** Diagram depicting for neutrophils (Ly6G^+^, CD11b^+^) in lungs of *Cdk14* Ctrl and *Cdk14* KO mice following co-injection of LPS and IFN-γ. Quantification about the number of neutrophils in the two groups. Cell frequencies show the percentage of target population in total cells. *Cdk14* Ctrl (IFN-γ + LPS) = 7；*Cdk14* KO (IFN-γ + LPS) = 6. Error bars, mean $\pm$ s.e.m. *P* values, *t*-test. **L** Diagram depicting for interstitial macrophages (CD64^+^) in lungs of *Cdk14* Ctrl and *Cdk14* KO mice following co-injection of LPS and IFN-γ. Quantification about the number of interstitial macrophages and monocytes in the two groups. Cell frequencies show the percentage of target population in total cells. *Cdk14* Ctrl (IFN-γ + LPS) = 7；*Cdk14* KO (IFN-γ + LPS) = 6. Error bars, mean $\pm$ s.e.m. *P* values, *t*-test. **M** Diagram depicting for B cells (B220^+^, CD19^+^) in lungs of *Cdk14* Ctrl and *Cdk14* KO mice following co-injection of LPS and IFN-γ. Quantification about the number of B cells in the two groups. Cell frequencies show the percentage of target population in total cells. *Cdk14* Ctrl (IFN-γ + LPS) = 7；*Cdk14* KO (IFN-γ + LPS) = 6. Error bars, mean $\pm$ s.e.m. *P* values, *t*-test.

In wildtype mice, co-injection of IFN-γ together with LPS significantly improves recovery of lung after injury. The overall morphology of lung was closer to undamaged lung in IFN-γ-treated mice (Fig. 6A). The area of EMCN^+^ blood vessel, NKX2.1^+^ and SFTPC^+^ epithelial cell was increased (Fig. 6B, C). In addition, IFN-γ treatment significantly alleviated the number of myeloid cells and neutrophil (Fig. 6D, E). The number of interstitial macrophage and monocyte was significantly reduced (Fig. 6F). Regarding lymphocyte, the number of B-lymphocyte returned to normal while CD4^+^ and CD8^+^ T-lymphocyte number was not changed (Fig. 6G and S6A). Therefore, IFN-γ treatment was very effective to improve recovery of lung during LPS-induced acute injury.

However, the tissue morphology of lung was not improved after IFN-γ treatment during LPS-induced injury in *Cdk14* knockout mice (Fig. 6H, I and S6B). The recovery of RAGE^+^ AT1 epithelial area, SFTPC^+^ AT2 epithelial cell number, NKX2.1^+^ epithelial cell number and Emcn^+^ vascular area were abolished (Fig. 6H, I). The EdU incorporation into lung was similarly reduced in *Cdk14* knockout mice (Fig. 6H). Regarding immune cells in the lung, the numbers of myeloid cells, neutrophil, interstitial macrophage, monocyte as well as B lymphocyte were dramatically higher in *Cdk14* knockout mice (Fig. 6J–M and S6C), reminiscence of LPS-induced lung injury without IFN-γ treatment (Fig. 3). These results indicated that IFN-γ-induced lung recovery was abolished in *Cdk14* knockout mice suggesting a potential crosstalk between CDK14 and IFN-γ signaling in vivo.

## Discussion

CDKs are essential regulator of cell cycle, but the function of individual CDK in different tissue remains to be explored. Recent scRNA-seq analysis across multiple organs in mammals sheds light on the understanding about organ-specific expression and function of CDKs, which indicated that many CDKs are not ubiquitously expressed in every organs [26]. For example, CDK5 is highly expressed in the brain. The brain, heart and most of internal organs are located within the mammalian body and are protected by multiple layers of skin, muscle, bone and the immune system, making the damage to these organs less likely to occur [27]. The respiratory system, however, is located outside the body and is highly susceptible to damage from the direct exposure to an external environment consisting of bacteria, viruses and potentially irritating gases. Therefore, our respiratory system possesses a certain degree of self-renewal and repair ability to deal with the exposure to the external environment. The regeneration and repair of lung is accompanied by cell proliferation and self-renewal, and the self-replication and division of these cells is precisely regulated by cell cycle protein-dependent kinases [28]. In this work, we find that CDK14 is indispensable for the regeneration of lung after bleomycin and LPS induced damage.

It has been shown that CDK14, as a cell cycle-dependent protein kinase, regulates the cell cycle transition from G2 to M phase mainly through the Wnt canonical signaling pathway. *Cdk14* has been reported to be enriched in malignant tumors, including triple-negative breast cancer. Therefore, targeting *Cdk14* provides a feasible method to block the progression of these tumors. However, most of these works rely on pharmacological reagent and shRNA, a precise genetic model to validate the function of CDK14 in vivo is lacking. Our work shows that genetic removal of exon 3 in *Cdk14* gene is an effective method to interrupt the function of this gene. Although it is unclear whether other CDK could compensate the effect of CDK14, this strategy to influence CDK14 function presents severe defects during embryonic development, steady-state maintenance in adult as well as lung regeneration induced by bleomycin and LPS. In addition to the epithelial and endothelial proliferative defects, we unexpectedly detected aberrant higher leukocyte ratio in the lung of *Cdk14* mice after both bleomycin and LPS induced damage. This is most likely because reduced endothelial proliferation cannot repair the pulmonary vascular barrier in time, resulting in prolonged inflammatory condition which is most likely harmful for the lung. Therefore, our work paves the way to utilize similar strategy to generate *Cdk14* flox conditional knockout mice in future to combine with cell type specific Cre mice to distinguish the function of this gene in different cell types and distinct organ in vivo.

As an important intracellular transcription factor, STAT1 is involved in the process of innate and acquired immune response in vivo, and has biological functions such as regulating the immune state of the body, anti-viral infection, anti-tumor [24], inhibiting the formation of fibrosis [29], and promoting the development of neurons [30]. IFN-γ phosphorylates when it binds to the receptor and activates STAT1, enhances the transcriptional activity of STAT1, and promotes the expression of downstream genes [31]. Indeed, there is scarce report about crosstalk between CDK family and IFN-STAT1 signaling. After nucleic acid sensing and virus infection, CDKs are necessary for the release of IFN-β [32]. CDK inhibition is reported to result in decrease of STAT2 phosphorylation at T387 [33]. A recent work reports that RANK overexpression in luminal breast cancer is associated with CDK4/6 inhibitor and decreased chronic IFN-γ response [34]. In our work, we provide evidence indicating that *CDK14* knockdown inhibits the transcription of *STAT1*, which, as far as we know, represents an unknown crosstalk mechanism between CDK family and IFN-STAT1 signaling. Our in vivo data argues that the crosstalk between CDK14 and IFN-γ is essential for the repair after lung injury and similar mechanism may be applied to other pathological and regenerative processes.

Together, our work illustrates the indispensable function of CDK14 in lung and identify the crosstalk between CDK14 and IFN-γ signaling as putative new target to promote lung repair.

## Materials and methods

### Animal experiments

C57BL/6 mice were used for all analysis of wild-type mice. Mice were typically sacrificed between 8 am and 10 am local time. Following overnight mating, female mice were examined in the following morning for the presence of a vaginal plug, which was counted as embryonic day 0.5 (E0.5). The developmental stage of embryos was confirmed by morphological features. Animals were housed in the animal facility of Guangzhou Institutes of Biomedicine and Health. Animal experiments were performed according to the institutional guidelines and laws, following the protocols (2024028) approved by local animal ethics committees.

*Cdk14* knockout mice (C57BL/6JGpt-*Cdk14*^em6Cd3867^/Gpt) were generated by GemPharmatech (Strain NO. T030463, Nanjing, China) and the third exon of the *Cdk14* gene was knocked out using CRISPR/Cas9 technology. In lung development experiments, FMF-04-159-2 was injected intraperitoneally at a dosage of 15 mg/kg consecutively for three days from E16.5 to E18.5. In lung regenerative repair experiments, 8–12 week-old C57BL/6 male mice were used as wild-type adult mice, and 50 μl of 0.3 mg/ml of bleomycin was used to construct a lung fibrosis model by airway injection, and 10 mg/kg of FMF was used to inhibit CDK14 by intraperitoneal injection after bleomycin. *Cdk14* knockout mice were constructed as a lung injury model by way of airway injection of 50 μl of 0.2 mg/ml of bleomycin. We induced acute lung injury by intratracheally injecting 4 mg/kg of LPS together with i.p. injecting 1 μg IFN-γ for three consecutive days into C57BL/6 male mice and *Cdk14* knockout mice.

### Cryosectioning, immunostaining and confocal imaging

Lungs were dissected and placed in ice-cold 4% paraformaldehyde (PFA-PBS) (Sigma, P6148) solution and fixed overnight. Fixed lung samples were dehydrated in 20% sucrose-PBS solution, and embedded in OCT (SAKURA, 4583) for storage at −80 °C. Cryosectioning was performed on a Leica CM3050 cryostat with low profile blades to achieve sections in the thickness of 10 µm. For immunostaining, pulmonary sections were rehydrated in PBS, permeabilized for about 15 min in 0.5% Triton X100-PBS solution and blocked for about 2 h in PBS with 1% BSA, 2% donkey serum, 10% Trion X100-PBS (blocking buffer) at room temperature. Sections were stained with primary antibodies diluted in blocking buffer at 4 °C overnight. After incubation, sections were washed three times with PBS and incubated with appropriate secondary antibodies diluted in blocking buffer at room temperature for 2 h. Nuclei were stained with DAPI during secondary antibody incubation. After that, sections were washed three times with PBS, mounted with Fluoromount-G (0100-01, Southern Biotech) and kept in 4 °C for confocal imaging. The primary antibodies include EMCN (Santa Cruz, sc-65495, 1:100), CD31 (R&D, AF3628, 1:100), RAGE (R&D, MAB1179-100, 1:100), NKX2.1 (Abcam, AB76013, 1:100), SFTPC (Merck, AB3786, 1:100), Car4 (R&D, AF2414, 1:100), αSMA (Thermo fisher, 50-9760-82, 1:100) were used in this study. The secondary antibodies include Donkey anti-Rat Alexa Fluor 488 (Thermofischer Scientific, A21208, 1:200), Donkey anti-Rabbit Alexa Fluor 546 (Thermofischer Scientific, A10040, 1:200), Donkey anti-Goat Alexa Fluor 647 (Thermofischer Scientific, A21447, 1:200), Sections were imaged with laser scanning confocal microscopes (LSM800) after immunohistochemistry. Pulmonary sections were imaged with the same microscope and imaging acquisition settings for quantitative analysis.

We used Fiji (open source; http://fiji.sc/) and Imaris (open source; https://imaris.oxinst.com/) for image processing in compliance with guide for digital images. In general, three technical replicate areas were randomly chosen from one biological sample for analysis. Images exported from Fiji were used to exhibition pictures.

### Histological staining

4% PFA fixed tissues were embedded in OCT (SAKURA, 4583) for storage at −80 °C. Cryosectioning was performed on a Leica CM3050 cryostat with low profile blades to achieve sections in the thickness of 14 µm, and stained with hematoxylin and eosin (Beyotime, C0105) or Sirius red stain (Solarbio, G1472).

### Flow cytometry

The left lung lobe was excised and minced for flow cytometry. The tissue was immersed in dissociation solution (5 ml 2% FCS-DMEM solution with 5 mg collage I (Gibco,17100017), 0.25 mg DNase I (Solarbio,D8071) and 3 U Dispase II (Sigma-Aldrich,42613-33-2) and incubated at 37 °C for 30 min. Samples were filtered using 100 μm Nylon cell strainer (Biosharp, BS-100-XBS) to get single cell suspensions, and cell centrifugation was performed at 300 g for 5 min. Red Blood Cell Lysis Buffer (Biolegend,420301) were diluted in deionized water and incubated with cells on ice for 10 min. Cells were washed by 2% FCS-PBS solution and then incubated with primary antibodies on ice for 30 min. Cells were washed again and resuspended in 2% FCS-PBS for flow cytometry. The following primary antibodies were used in this study. CD45-Pacific Blue (Biolegend,30-F11,103126), CD45R(B220)-APC (Biolegend,RA3-6B2,103212), CD19-FITC (Biolegend,6D5,115506), CD11b-FITC (Biolegend,M1/70,101206), anti-Gr-1-APC (Biolegend,RB6-8C5,108412), CD8a-APC (Biolegend,QA17A07,155006), anti-CD4-FITC (Biolegend,RM4-5,100510), CD11c-Pacific Blue (Biolegend,N418,117322), anti-F4/80-APC-eFluor780 (Invitrogen,BM8,47-4801-82), CD11b-Percp-Cy5.5 (Biolegend,M1/70,101228), CD3-PE (Biolegend,17A2,100206), CD64-PE (Biolegend,X54-5/7.1,139304), Ly6G-PE (Biolegend,1A8,127608), CD45-PE-Cy7 (Biolegend,G8.8,118216).

### Cell culture

The human umbilical vein endothelial cells (HUVECs) were purchased from ATCC (PCS-100-013), cultured in ECM medium (ScienCell,1001) with extra 10% Fetal Bovine Serum (FBS) (NEWZERUM, FBS-E500) and grown in 5% CO_2_ at 37 °C. A549 cell line was cultured in DMEM medium (Gibco, C11995500BT) with 10% FBS and 1% P/S. HEK293T/17 cells were seeded in DMEM in 10 cm plates at 50% confluency. The cells were transiently co-transfection with lentiviral with pLL3.1-GFP-shCDK14/PAX2/pVSVG.

### Immune cell recruitment assay

bEnd.3 cells were infected with shCtrl or shCDK14 lentivirus for 72 h in DMEM medium with 10% FBS and 1% P/S. A total of 3 × 10^4^ bEnd.3 cells/well of 8-mm transwells (Corning Life Sciences), coated with 100 µg/mL Fibronectin (Yeasen, 40113ES03), were grown for 16 h and stimulated 4 h before the assay with 10 ng/mL TNF-α (Peprotech, 315-01 A). After washing away the cytokine, 1 × 10^6^ FACS-sorted bone marrow-derived myeloid cells (CD45^+^ CD11b^+^) were added per transwell, and 100 ng/mL CXCL12 (Peprotech, 250-20a) was added in the bottom chamber. Transmigration was allowed for 2 h. Migrated myeloid cells were collected for cell counting. Myeloid cells were fixed using 4% paraformaldehyde. Subsequently, the cells were stained with Crystal violet (Beyotime, C0121) for 30 min, cleaned with PBS, and photographed using an inverted fluorescence microscope (Olympus IX73) for the purpose of quantification.

After intratracheally injecting of LPS, we exposed *Cdk14* Ctrl or *Cdk14* KO recipient mice to 2.5 Gy γ-irradiation before transplantation. We isolated bone marrow cells from *Vav1-Cre Rosa26-mTmG* transgenic mice and injected recipient mice with 1 × 10^7^ bone marrow cells via tail vein injection. We analyzed the GFP^+^ donor hematopoietic cells (GFP^+^ CD45^+^ or GFP^+^ CD45^+^ CD11b^+^) 16 h after bone marrow cell transfer.

### Transwell invasion assay

Treated HUVECs were resuspended in serum-free culture medium and their concentration was adjusted to 1 × 10^4^ cells/ml. A volume of 200 μl of the cell suspended was introduced into the upper chamber, while 700 μl of cell culture medium containing 10% FBS was added to the lower chamber. The cells were then incubated in a 37 °C and 5% CO_2_ incubator for 16 h. Following this, they were fixed with 70% ethanol for 30 min. Subsequently, the cells were stained with Crystal violet (Beyotime, C0121) for 30 min, cleaned with PBS, and photographed using an inverted fluorescence microscope (Olympus IX73) for the purpose of counting. Each group was allocated three multiple wells, and for each well, three fields of view were randomly selected at a magnification of 4×. This was done to quantify the average number of invading cells.

### Scratch assay

The A549 cells with lentivirus was used when reaching 90% confluence in 24-well plates. A clean scratch across the center of the cell layer was generated using a scratcher (SPL life sciences, 201924). Photographs were taken at 0 and 48 h using an inverted fluorescence microscope, and cell migration distance was estimated by Fiji (open source; http://fiji.sc/).

### 5-Ethynyl-2’-deoxyuridine (EdU) staining

The cultured cells were labelled with EdU, fixed, washed, and permeabilized following manufactured protocol (APE$\times$BIO, K1078). Click reaction solution was used for the EdU reaction and detection, fluorescence was observed under a flow cytometry.

### PI staining assay

Treated HUVEC and A549 cells were harvested by trypsinization and centrifuged at 500 g. Then cells were fixed in pre-cooling 70% ethanol overnight. After washed twice with PBS, cells were mix with PI staining solution (Beyotime, ST008) for 30 min at 37 °C. The flow cytometry was used to detect the red fluorescence.

### RNA-seq sample preparation and data processing

HUVEC and A549 cells were collected into a centrifuge tube using 500 μl of Trizol after washing away the residual medium with PBS. After extracting RNA, the library preparation and sequencing was performed following instruction from Illumina. The raw sequence data from Bulk RNA-seq were quality-trimmed using the default options of fastp software v0.23.4. Reads were aligned to the human genome hg19 using Bowtie2 v2.4.4. Transcript abundance was measured with RSEM v1.3.1. Custom scripts written in R v4.2.2 were used to perform the analysis. Differential gene expression across sample pairs was evaluated using DESeq2 v1.38.0, focusing on Fold Change (FC) and p-values for multiple hypothesis testing. In the sh*Cdk14* group, 70 and 384 genes showed significant differential expression (log2 FC > 0.5, *p*-value < 0.05), compared to 161 and 556 genes in the control group, in both HUVEC and A549 cells. Visualization of differentially expressed genes was performed using Pheatmap v1.0.12. Gene ontology analysis was conducted using Kobas v3.0; common results between the two cell types with a *p*-value < 0.05 were selected, and visualization was done using circlize v0.4.15. We used VennDiagram v1.7.3 to show 93 common differentially expressed genes between the two cell types. Further visualization, including volcano plots, was performed using ggplot2 v3.4.0.

### Quantification and statistics

All results are presented as mean ± s.e.m. Number of animals or cell samples represents biological replicates and is indicated in corresponding figure legends. Mice that died before the completion of experimental protocols were excluded from analysis, which was a pre-established criterion before the experiment. The animals were allocated based on genotyping results; therefore, no randomization and blinding were used. Comparisons between different groups were performed using two-tailed Student’s *t* test unless otherwise indicated in figure legends. No statistical methods were used to predetermine sample size. The animal sample size was determined based on experience in this research field. Before the Student’s *t*-Test, samples from different groups were tested using *F*-test to identify the variances between groups. *F* value less than 0.05 indicated samples have significantly different variances. *P* values are indicated in the graphs and *P* values below 0.05 were considered to be statistically significant. The original sequencing data were available in China National Center for Bioinformation (PRJCA027249). We have no computer code that are central to the conclusion.

## Supplementary information

Supplementary Figures and legends

## Data Availability

The data used in this study are available from the corresponding author upon reasonable request.
