# Supplementary material for: CDK14 regulates the development and repair of lung
Source: Cell Death Discov. 2025 Jan 18;11:12. doi: 10.1038/s41420-025-02292-4 (PMC11743204; doi:10.1038/s41420-025-02292-4)
Supplement: Supplementary file 1 — Supplementary Figures and legends [file 41420_2025_2292_MOESM1_ESM.pdf]

**Supplementary Fig.1. The expression pattern of CDK14.**

(A) *Cdk14* gene knockout strategy.

(B) Genotype statistics of offspring from *Cdk14* heterozygous knockout parents.

(C) *Cdk14* expression in pulmonary cells of mice.

(D) *Cdk14* expression in different organs of mice.

(E) Representative projected confocal images showing $IB4^+$ (red) cerebral vasculature and $DCX^+$ (blue) neural progenitor cells in *Cdk14* Ctrl or *Cdk14* KO E19.0 brain. Quantification about average intensity and covered area of $IB4^+$ blood vessels and intensity of $DCX^+$ Cells. All data are normalized to *Cdk14* Ctrl. *Cdk14* Ctrl =3; *Cdk14* KO =3. Error bars, mean ± s.e.m. *P* values, *t*-test.

**Supplementary Fig.2. The epithelial and endothelial defects in adult *Cdk14* knockout mice.**

(A) Representative histological morphology of hematoxylin-eosin (left panel) and sirius red (right panel) staining of lung from *Cdk14* Ctrl and *Cdk14* KO steady-state adult mice.

(B) Representative projected confocal images showing EMCN+ (green) blood vessels and NKX2.1+ (orange) lung epithelial cell from *Cdk14* Ctrl and *Cdk14* KO steady-state adult mice. Quantification about area of EMCN and NKX2.1 labelled lung epithelial cell in *Cdk14* Ctrl and *Cdk14* KO mice lung. All data are normalized to *Cdk14* Ctrl. *Cdk14* Ctrl = 4； *Cdk14* KO = 3. Error bars, mean ± s.e.m. *P* values, *t*-test.

(C) Representative projected confocal images showing RAGE+ (green), SFTPC+

(orange) and CD31 (red) cells from *Cdk14* Ctrl and *Cdk14* KO steady-state adult mice. Quantification about RAGE$^+$ area and SFTPC-labelled AT2 cell number from *Cdk14* Ctrl and *Cdk14* KO steady-state adult mice. All data are normalized to *Cdk14* Ctrl. *Cdk14* Ctrl = 4； *Cdk14* KO = 4. Error bars, mean ± s.e.m. *P* values, *t*-test.

**Supplementary Fig.3. CDK14 inhibitor FMF-04-159-2 induced abnormal lung regeneration of after bleomycin treatment.**

(A) Representative histological morphology of Vehicle and FMF mice lung after bleomycin treatment stained by hematoxylin-eosin and sirius red.

(B) Representative projected confocal images showing EMCN$^+$ (green) blood vessels and NKX2.1$^+$ (blue) lung epithelial progenitor cell and αSMA (red) labelled myofibroblasts in bleomycin-treated Vehicle and FMF mice lung. Quantification about covered area of EMCN and αSMA and NKX2.1 labelled lung epithelial progenitor cell number in bleomycin-treated Vehicle and FMF mice lung. All data are normalized to Vehicle. Vehicle = 4； FMF = 4. Error bars, mean ± s.e.m. *P* values, *t*-test.

(C) Representative projected confocal images showing RAGE$^+$ (green), SFTPC$^+$ (blue) and DAPI$^+$ cells in bleomycin-treated Vehicle and FMF mice lung. Quantification about intensity of RAGE and SFTPC cell number in bleomycin-treated Vehicle and FMF mice lung. All data are normalized to Vehicle. Vehicle = 3； FMF = 3. Error bars, mean ± s.e.m. *P* values, *t*-test.

**Supplementary Fig.4. CDK14 removal influences immune cell recruitment.**

(A) Diagram depicting for $CD4^+$ ($CD3^+$, $CD8^-$, $CD4^+$) and $CD8^+$ ($CD3^+$, $CD8^+$, $CD4^-$) cells in LPS-treated *Cdk14* Ctrl and *Cdk14* KO mice lung. Quantification about the number of $CD4^+$ and $CD8^+$ cells in the two groups. Cell frequencies show the percentage of target population in total cells. *Cdk14* Ctrl = 5; *Cdk14* KO = 4. Error bars, mean ± s.e.m. *P* values, *t*-test.

(B-C) Diagram depicting percentage of $GFP^+$ $CD45^+$ (B) or $GFP^+$ $CD45^+$ $CD11b^+$ (C) cell number in *Cdk14* Ctrl or *Cdk14* KO host mice lung after sublethal irradiation and transplantation of $GFP^+$ donor hematopoietic cells. Quantification about the number of $GFP^+$ $CD45^+$ cells in the two groups. Cell frequencies show the percentage of target population in total cells. *Cdk14* Ctrl = 3; *Cdk14* KO = 3. Error bars, mean ± s.e.m. *P* values, *t*-test.

(D) Diagram illustrates myeloid trans-endothelial cell migration assay *in vitro*.

(E) Representative image and quantification of myeloid cell number trans-migrate endothelial cell barrier formed by shCtrl (n=4) or shCDK14 (n=4) endothelial cells. Error bars, mean ± s.e.m. *P* values, *t*-test.

**Supplementary Fig.5. CDK14 ablation influences cell migration and proliferation.**

(A) RT-PCR showing the intracellular mRNA levels of *Cdk14* in HUVEC treated with shCDK14. All data are normalized to shCtrl. shCtrl = 3; shCDK14-1= 3; shCDK14-2 = 3. Error bars, mean ± s.e.m. *P* values, *t*-test.

(B) RT-PCR showing the intracellular mRNA levels of *Cdk14* in bEnd.3 treated with shCDK14. All data are normalized to shCtrl. shCtrl = 3; shCDK14 = 3. Error bars, mean

± s.e.m. *P* values, *t*-test.

(C) bEnd.3 were subjected to cell counting after infected with shCDK14 to knockdown. All data are normalized to shCtrl. shCtrl = 6; shCDK14 = 6. Error bars, mean ± s.e.m. *P* values, *t*-test.

(D) bEnd.3 were infected with shCDK14 to knockdown *Cdk14*, after infection, transwell assays were performed, and pictures were taken 16 h later. All data are normalized to shCtrl. shCtrl = 7; shCDK14 = 7. Error bars, mean ± s.e.m. *P* values, *t*-test.

**Supplementary Fig.6. CDK14 does not influence T lymphocyte during LPS-induced repair.**

(A) Diagram depicting for $CD4^+$ ($CD3^+$, $CD8^-$, $CD4^+$) and $CD8^+$ ($CD3^+$, $CD8^+$, $CD4^-$) cells in lungs from mice with LPS induced lung injury, treated with PBS or IFN-γ. Quantification about the number of $CD4^+$ and $CD8^+$ cells in the two groups. Cell frequencies show the percentage of target population in total cells. WT (PBS + LPS) = 5; WT (IFN-γ + LPS) = 6. Error bars, mean ± s.e.m. *P* values, *t*-test.

(B) Representative histological morphology of lungs of *Cdk14* Ctrl and *Cdk14* KO mice following co-injection of LPS and IFN- γ and stained by hematoxylin-eosin.

(C) Diagram depicting for $CD4^+$ ($CD3^+$, $CD8^-$, $CD4^+$) and $CD8^+$ ($CD3^+$, $CD8^+$, $CD4^-$) cells in lungs of *Cdk14* Ctrl and *Cdk14* KO mice following co-injection of LPS and IFN-γ. Quantification about the number of $CD4^+$ and $CD8^+$ cells in the two groups. Cell frequencies show the percentage of target population in total cells. *Cdk14* Ctrl (IFN-γ

+ LPS) = 7; *Cdk14* KO (IFN-γ + LPS) = 6. Error bars, mean ± s.e.m. *P* values, *t*-test.

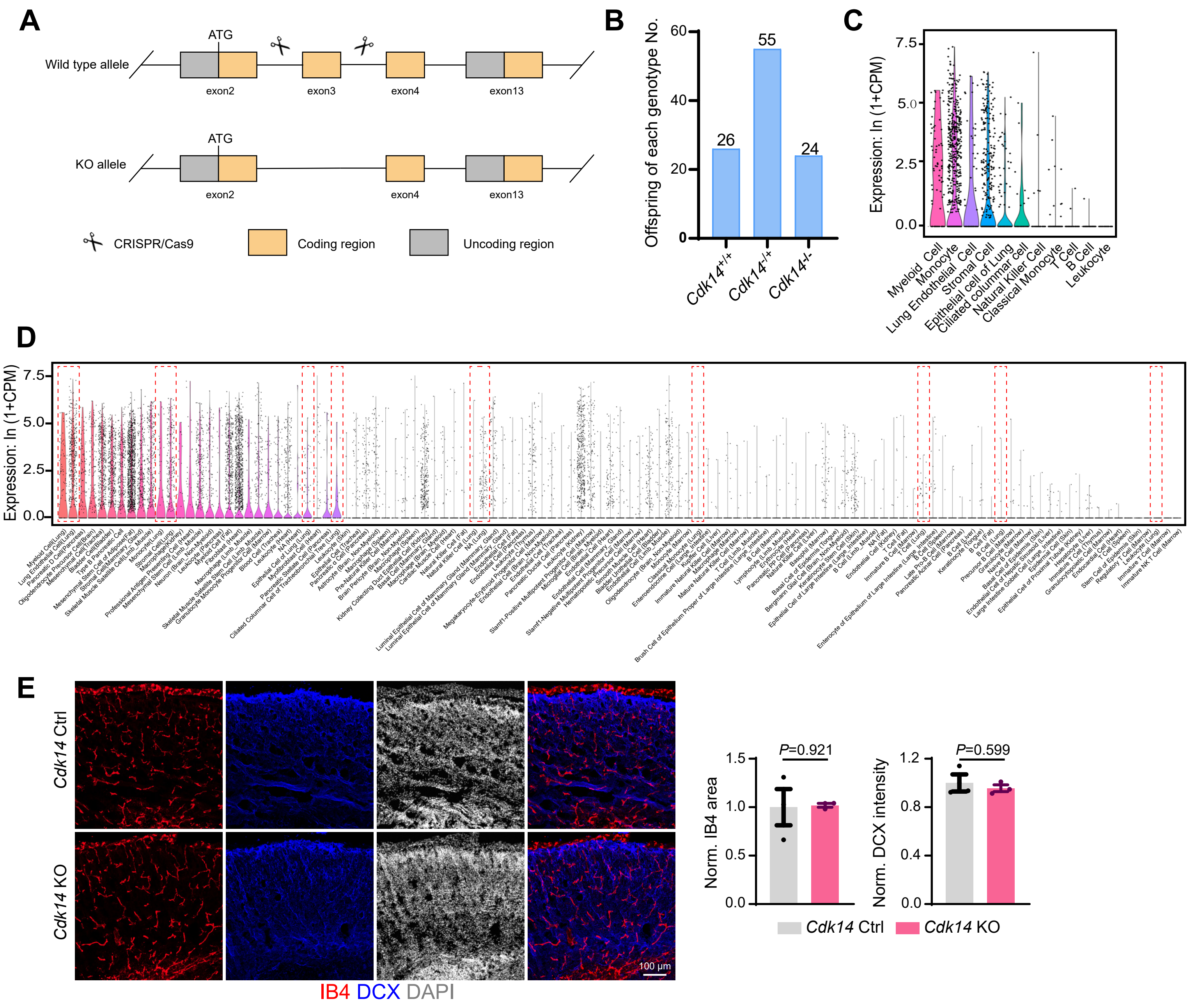


Supplementary Figure 1. Chen et al.

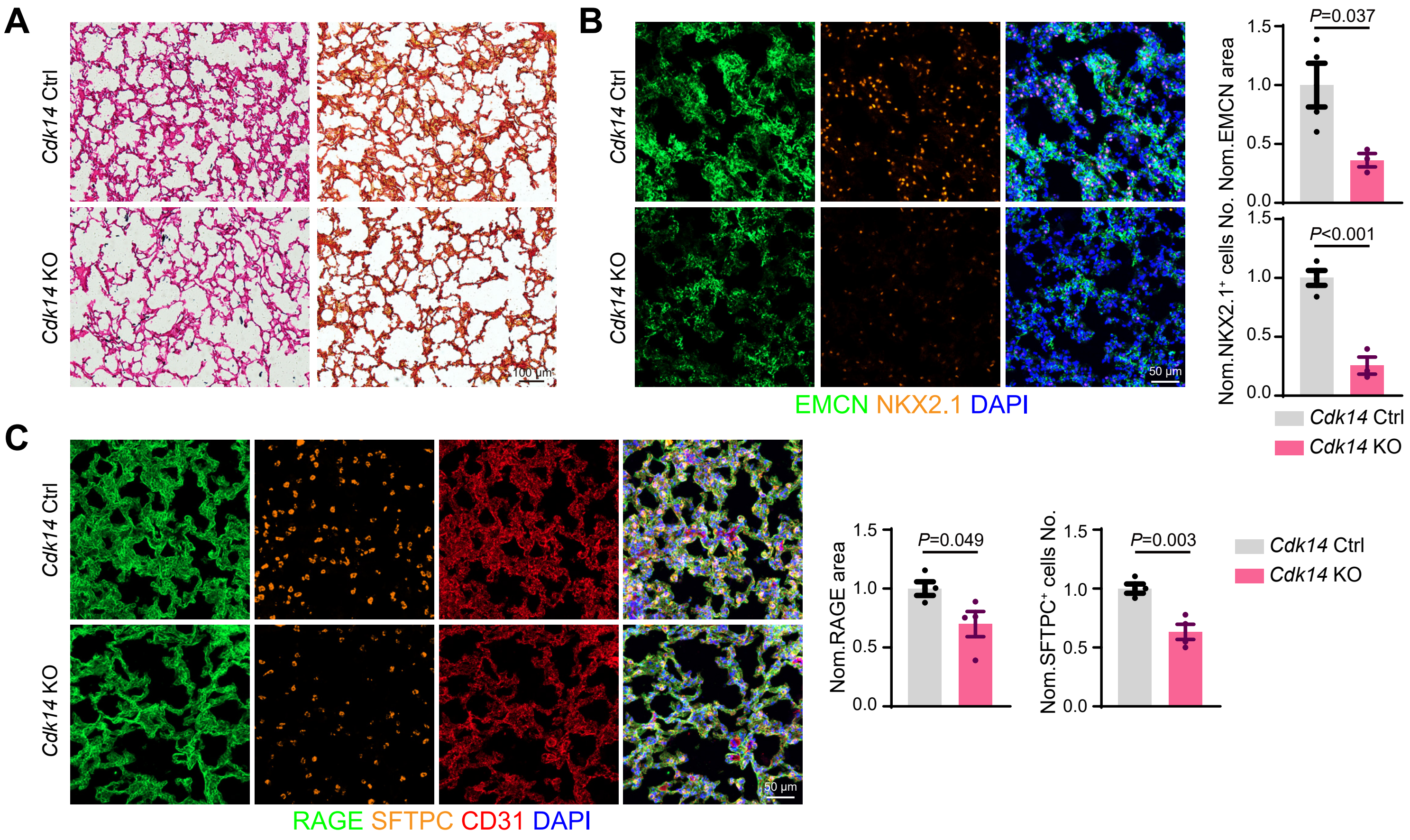


Supplementary Figure 2. Chen et al.

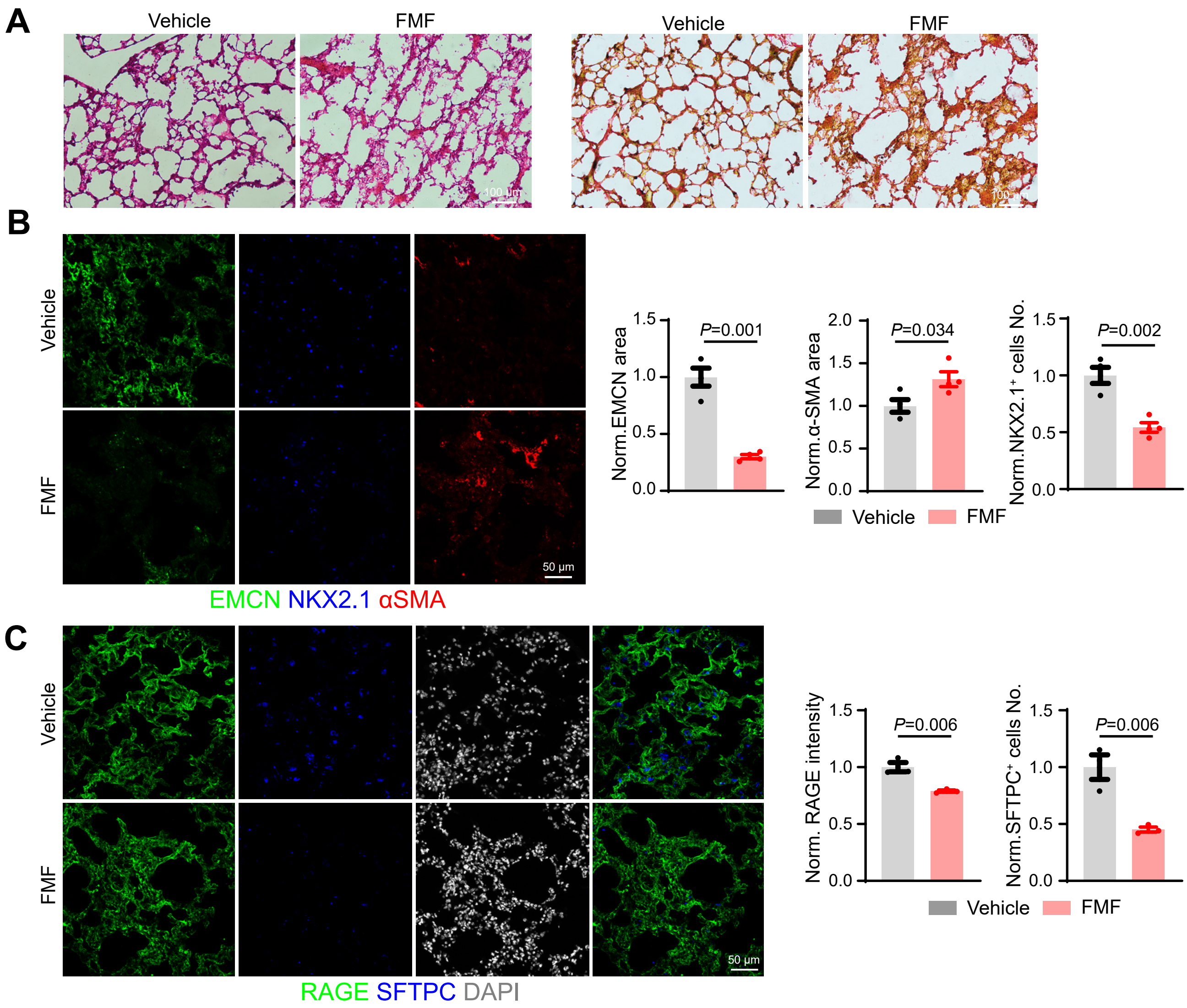


Supplementary Figure 3. Chen et al.

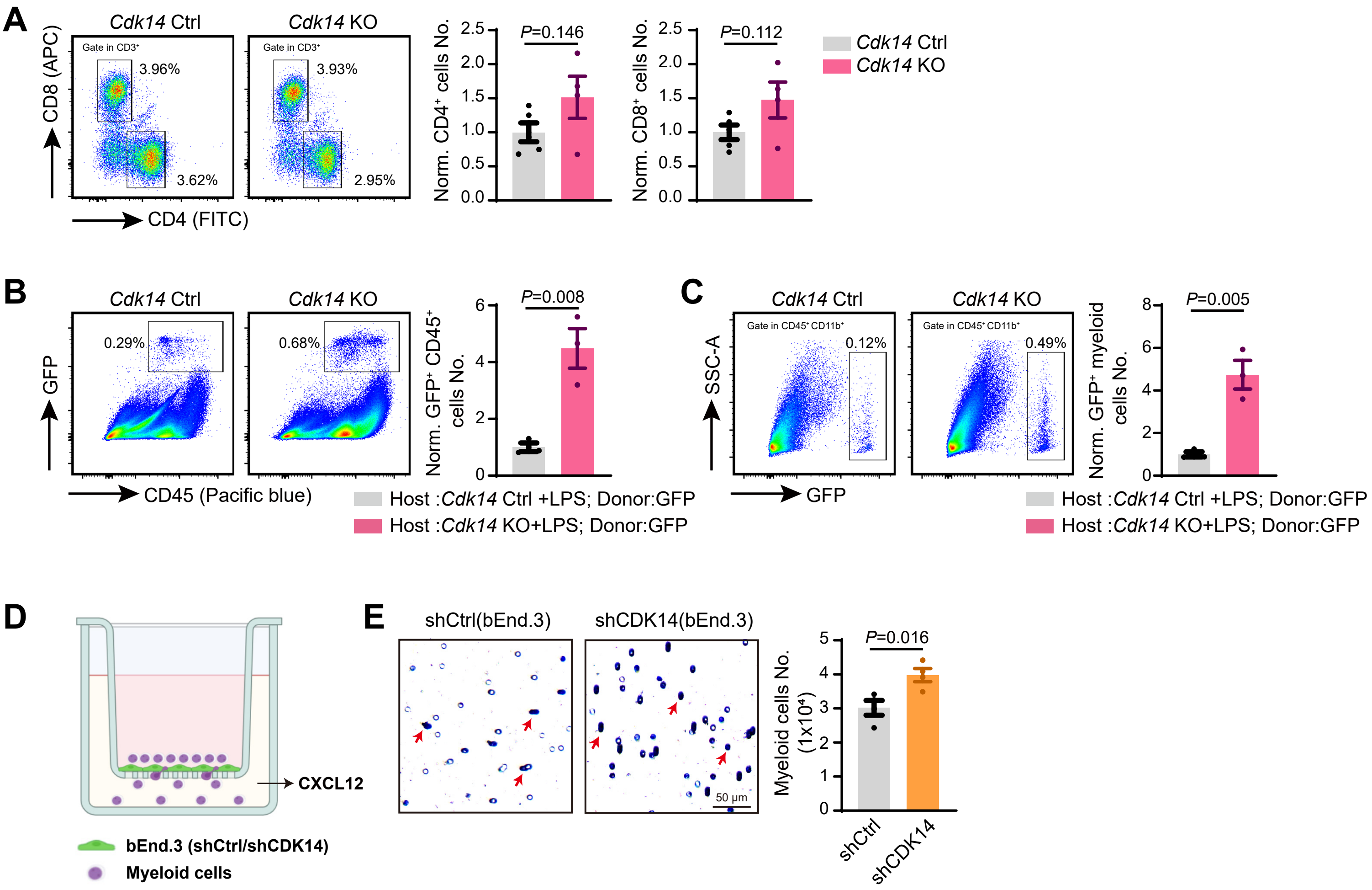


Supplementary Figure 4. Chen et al.

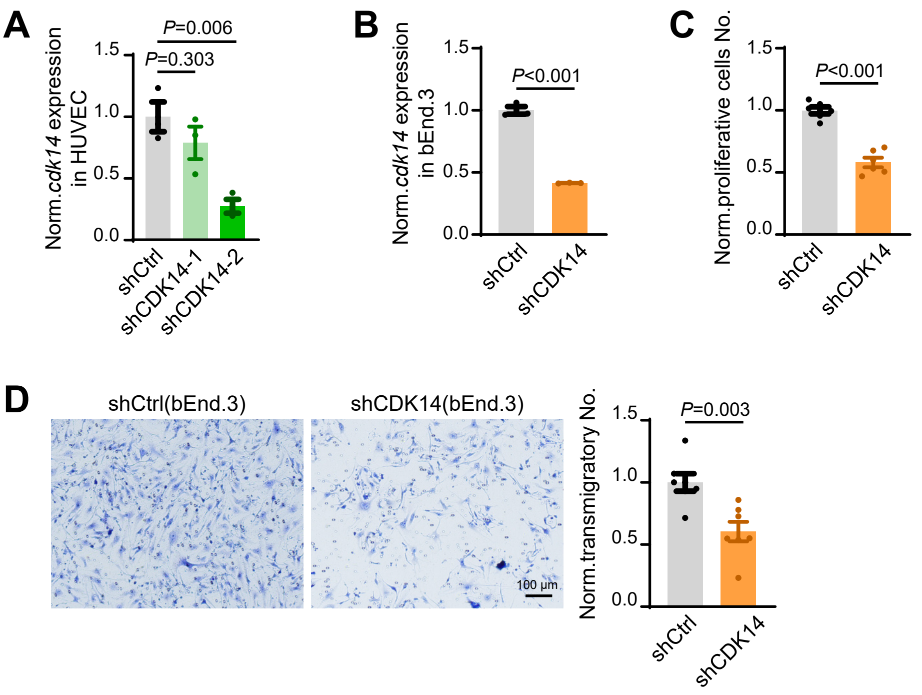


Supplementary Figure 5. Chen et al.

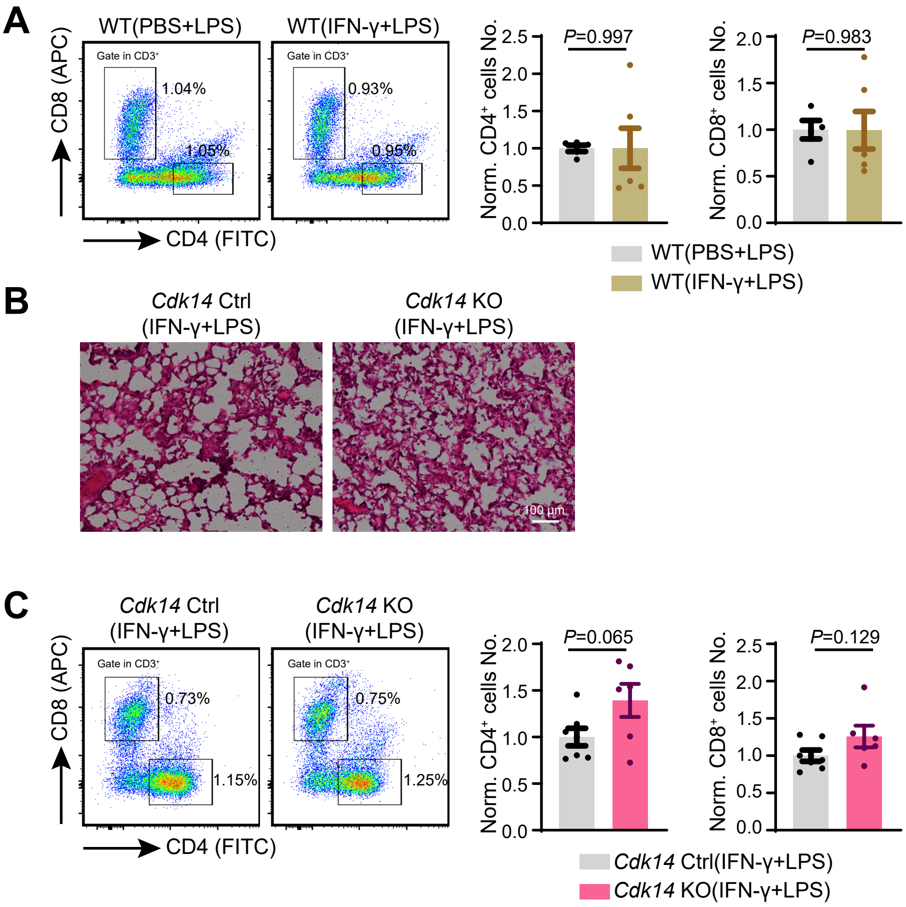
A
WT(PBS+LPS)
WT(IFN-γ+LPS)
Gate in CD3+
1.04%
1.05%
0.93%
0.95%
CD8 (APC)
CD4 (FITC)
Norm. CD4+ cells No.
P=0.997
Norm. CD8+ cells No.
P=0.983
WT(PBS+LPS)
WT(IFN-γ+LPS)
B
Cdk14 Ctrl (IFN-γ+LPS)
Cdk14 KO (IFN-γ+LPS)
100 µm
C
Cdk14 Ctrl (IFN-γ+LPS)
Cdk14 KO (IFN-γ+LPS)
Gate in CD3+
0.73%
1.15%
0.75%
1.25%
CD8 (APC)
CD4 (FITC)
Norm. CD4+ cells No.
P=0.065
Norm. CD8+ cells No.
P=0.129
Cdk14 Ctrl(IFN-γ+LPS)
Cdk14 KO(IFN-γ+LPS)
